# Comparison Between Pregabalin and Sertraline in the Treatment of Uremic Pruritus in Patients With Maintenance Hemodialysis

**DOI:** 10.7759/cureus.65788

**Published:** 2024-07-30

**Authors:** Irshad A Ansari, Muhammad A Anees, Najim Sekh, Aasma Urooj

**Affiliations:** 1 Nephrology, King Edward Medical University, Lahore, PAK; 2 Animal Science, Himalayan College of Agricultural Sciences and Technology, Kathmandu, NPL; 3 Dialysis, King Edward Medical University, Lahore, PAK

**Keywords:** comparison, 5d score scale, maintenance hemodialysis, uremic pruritus, sertraline, pregabalin

## Abstract

Introduction

Pruritus, a medical symptom also known as itch, is characterized by an unpleasant feeling coupled with the urge to scratch. Patients with chronic kidney disease (CKD) often have symptoms of pruritus due to uremia, which has adversely affected their quality of life. The treatment protocol for CKD pruritus is still being debated due to its unclear etiopathogenesis. Pregabalin, an anticonvulsant medication, and sertraline, an antidepressant medication, have been used to treat uremic pruritus (UP) patients on maintenance hemodialysis.

Objectives

There are no adequate investigations comparing the efficacy of pregabalin and sertraline, particularly in under-resourced countries such as Pakistan.

Method

The research was a randomized trial for a period of four weeks at the nephrology department of the Mayo Hospital, Lahore. A total of 62 patients were randomly assigned to take either pregabalin (aged 48.06±13.44) or sertraline (aged 47.45±10.97) tablet once a day, a total of 31 patients to each group. The pregabalin group was specified 25 mg for the first week, 50 mg for the second week, and 75 mg for the third and fourth week. Similarly, sertraline was prescribed 25 mg for the first week and 50 mg for the remaining three weeks. However, if the patient shows improvement on the lowest dosage, therapy with the same minimum dose was proposed to be continued. Lastly, the itching score was assessed on the 5D pruritus scale. The Visit 1 (no drug) score was evaluated against post-therapy scores at two-week intervals as Visit 2 (week 2) and Visit 3 (week 4). Statistical analysis was done using the Statistical Product and Service Solutions (SPSS, version 26; IBM SPSS Statistics for Windows, Armonk, NY) with a significance level set at p<0.05 and 95% confidence level.

Conclusion

This research concluded that both pregabalin and sertraline significantly improved itching intensity in each treatment group; however, there was no significant difference between the two drugs in reducing UP based on the 5D itching scores. In each domain of the 5D pruritus scale, a significant difference was found in post-follow-up pregabalin and sertraline therapy.

## Introduction

The kidneys are essential in regulating body temperature and acid-base balance. Loss of kidney function leads to difficulties in almost every organ system due to the loss of excretory, regulatory, and endocrine function [1]. If the estimated glomerular filtration rate (eGFR) is less than 60 mL/min/1.73 m^2^, the patient will be considered to have chronic kidney disease (CKD), which is characterized by decreased kidney function for more than three months [2]. About 10%-14% of the population has CKD in Pakistan, with nearly 21 million Pakistanis found to have CKD stage 3 or 4 based on a study conducted in 2005-2006 [3]. Age, hypertension, diabetes mellitus, cardiovascular disease, kidney stones, recurrent urinary tract infection (UTI), autoimmune illnesses, use of nephrotoxic medicines, obesity, systemic infections, decreased renal mass, and prior acute kidney damage are risk factors for CKD [4]. One symptom of CKD is uremic pruritus (UP). It is an annoyance that makes the sufferer scrape his/her own skin. It is still one of the most annoying problems that lead to wrinkled skin, restless nights, low mood, anxious thoughts, and, overall, worse quality of life. The pattern of UP changes in individuals with renal illness. Even though the back, chest, and extremities tend to be commonly affected, anywhere from 20% to 40% of people may have widespread pruritus [5].

UP in CKD patients has no definitive explanation, but it is believed to be linked with secondary hyperparathyroidism and elevated levels of blood minerals and substance P [5]. Other risk factors for UP include xerosis, anemia, peripheral neuropathy, chronic inflammatory diseases, and a high incidence of human leukocyte antigen (HLA) B35 [6]. Thus, several pharmaceutical and natural remedies have been proposed for the treatment of pruritus caused by end-stage renal disease (ESRD), including anticonvulsants (pregabalin, gabapentin), antidepressants (sertraline, mirtazapine, paroxetine, and doxepin), opioids, ultraviolet B-light (UVB), and homeopathic food such as turmeric [7].

In this research, pregabalin and sertraline drugs were compared as a randomized trial because very limited research has been conducted previously on their efficacy both regionally and globally. Pregabalin has been shown in several trials to improve hemodialysis patients' quality of life and to decrease the intensity of adverse symptoms without causing any severe side effects [8-11]. It is believed to act by inhibiting neurotransmitters, which trigger itching by blocking glutamate release before a synaptic gap, originally designed as a gamma-aminobutyric acid (GABA) mimetic. The release of neurotransmitters, such as noradrenaline, glutamate, and substance P, is hypothesized to be decreased by pregabalin [12]. Additionally, pregabalin has been shown to decrease the production of calcitonin and gene-related peptide, which are two mediators of itching. Numerous trials have shown the effectiveness of pregabalin in treating UP patients significantly. For instance, pregabalin demonstrated a quick and efficient response in reducing pruritus in nearly 60% of participants in research done in Iran [10].

Sertraline, on the other hand, has been shown to inhibit selective serotonin reuptake and acts as an anti-depressant therapeutic choice [13]. In addition to being effective in treating uremic pruritus, sertraline was formerly used to treat cholestatic pruritus. According to some reports, serotonin stimulates nociceptive nerve fibers. Thus, it is believed that serotonin receptors may also be involved in the itch signal since it causes itch in humans when injected or administered by iontophoresis [14]. Furthermore, sertraline may be prescribed to individuals with ESRD without requiring a dosage change [15]. When alternative treatments fail to alleviate uremic pruritus, sertraline may be tried, particularly among the subset of dialysis patients who also suffer from depression and need antidepressant medication [16]. Serotonin receptors affect the transmission of opioid-inhibitory signals in the brain. This is how it is believed that sertraline, a serotonin receptor inhibitor, may lessen pruritus in stage-V patients with chronic renal disease [17]. Nevertheless, constipation, sleeplessness, headaches, and gastrointestinal toxicity are common adverse effects of sertraline. It is believed that anti-inflammatory medications such as sertraline may reduce the levels of inflammatory markers and cytokines [13].

To sum up, the research was directed to examine the effectiveness of anticonvulsant pregabalin and antidepressant sertraline in treating uremic pruritus patients on maintenance hemodialysis. Thus far, there has been no adequate regional, national, and global research conducted on this topic. Therefore, there is a paucity of information indicating which of these two medications is appropriate based on their availability, particularly in under-resourced medical settlements.

## Materials and methods

**Figure 1:**
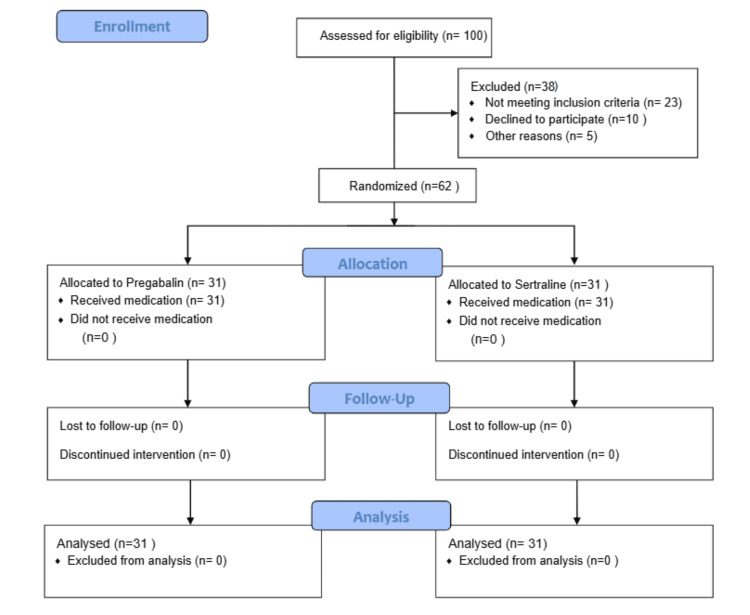
Consolidated Standards of Reporting Trials (CONSORT) flow diagram of the clinical trial

The study was conducted as a randomized trial to assess the efficacy of pregabalin and sertraline in patients under maintenance hemodialysis, as shown in Figure 1. A simple lottery method was used to choose patients at the nephrology department of the Mayo Hospital, Lahore, Pakistan, spanned from the third of December 2020 to the first of June 2021, providing an adequate timeframe to collect, evaluate, and interpret data from dialysis patients of indoor and outdoor settings. Inclusion criteria encompassed individuals aged 15-70 years under maintenance hemodialysis (twice weekly) for the last three months to 10 years of clinical history who were unresponsive to antihistamines, anti-inflammatory drugs, or topical lubricants. Meanwhile, exclusion criteria included patients with clinical manifestations such as scabies, obstructive jaundice, and hematological pruritus. In addition, cancer patients in active treatment, expectant and nursing mothers, and thyroid patients - hypothyroidism/hyperthyroidism - were precluded.

This study adhered to the strict ethical standards, having received approval from the Board of Studies in Nephrology (Neph/101/2019), Project Evaluation Committee (1174/PEC/RC/KEMU), Institutional Review Board (28/RC/KEMU), and Advanced Studies and Research Board (7827/KEMU/2020) of the King Edward Medical University (KEMU)/Mayo Hospital, Lahore. The national clinical trial registration number is 22061/REG/KEMU/2020. Informed consent was obtained from all participants before the research. The required sample size was calculated using the basic formula for difference in means. Based on the assumed significance level of 5%, the power of the test at 90%, and the predicted mean value as the dependent variable, the sample size for this research was calculated to be 62 patients. Thus, the eligible cohort, which comprised a total of 62 patients, was randomly assigned to take either pregabalin or sertraline: 31 patients in each group.

Table 1 compares two groups, pregabalin and sertraline, with respect to age and gender. The mean ages are similar (48.06 vs 47.43), and gender distribution is comparable with males higher than females in both study groups. These findings are supported by p-values of 0.845 and 1, respectively, suggesting that the two groups are well-matched for clinical trials.

**Table 1:** Demographical data distribution among research groups P-value of less than 0.05 is considered statistically significant.

Variables		Pregabalin Group (No. 31)	Sertraline Group (No. 31)	P-value
Age	Mean±SD	48.06±13.44	47.43±10.97	0.845
	Min	24	24	
	Max	80	70	
Gender	Male	20 (64.5%)	21 (67.7%)	1
	Female	11 (35.5%)	10 (32.3%)	

The 5D itch scale, a simple - yet comprehensive - questionnaire for measuring UP was employed where degree, time, orientation, impairment, and spatial distribution were the five factors under investigation, as exhibited in Figure 3. The total 5D score was calculated by adding the individual domain scores. The possible range of the 5D score is from five (no pruritus) to 25 (extreme pruritus). Scores of 5-9 indicate the absence of pruritus, 10-14 indicate mild pruritus, 15-19 indicate moderate pruritus, and 20-25 indicate severe pruritus [18]. The number shown underneath each answer option is the score for that domain on a single item in terms of time, intensity, or orientation (range: 1-5). The effects of itching on four major facets of daily life-sleep, leisure/social activities, housework/errands, and work/school were measured by four items in the disability domain. This examination used the highest possible score from any of the four elements to determine the disability domain score. The distribution domain score was calculated by adding up the number of afflicted body parts (possible total: 0-16) and then assigning points accordingly (0-2) = 1, (3-5) = 2, (6-10) = 3, (11-13) = 4, and (14-16) = 5.

Lastly, the research data were analyzed by the Statistical Product and Service Solutions (SPSS, version 26; IBM SPSS Statistics for Windows, Armonk, NY) software, where the T-test was used among the group, comparing the score before drug therapy (Visit 1) and post follow-ups at Visit 2 and Visit 3. Meanwhile, the chi-square test was used to compare each domain of pruritus intensity for the pregabalin and sertraline groups, comparing the 5D score before drug therapy (Visit 1) and post follow-ups after medication at Visit 2 and Visit 3. The significant level was set at a p-value of <0.05 with a confidence level of 95%.

**Figure 2:**
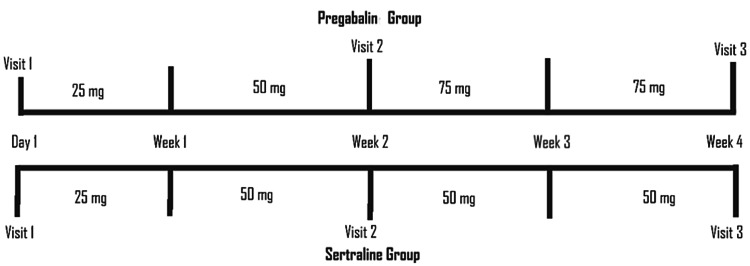
Proposed drug therapy timeline

Figure 2 presents the proposed medication plan for pregabalin and sertraline groups at three consecutive visits on a weekly basis. Group members receiving pregabalin were prescribed 25 mg tablets once daily for the first week, 50 mg tablets once daily for the second week, and 75 mg tablets once daily for the third and fourth weeks. Similarly, sertraline was prescribed 25 mg tablet once daily for the first week and 50 mg tablet once daily for the remaining three weeks. The Urdu version of the 5D-itching scale for assessing pruritus was employed for patients' convenience. However, if the patient shows improvement while on the lowest dosage, therapy with the same minimum dose was proposed to be continued for the remaining study period. After assessing the initial 5D itching score (Visit 1 - no drug), the patients were followed up at intervals of two weeks post therapy: Visit 2 (week two) and Visit 3 (week four).

## Results

The proposed medication plan was adjusted based on the clinical outcome of patients in this research, as shown in Table 2. Firstly, the patients in both groups were given 25 mg of either drug after initial screening of the 5D pruritus score before drug therapy. After two weeks, 30 (96.8%) patients in both groups were given 50 mg of either drug, and only one (3.2%) patient in each group had given a previous dose of 25 mg. However, till the fourth week post treatment, patients in both groups were given a 50 mg dose of medications. At this point in time, none of the patients in either treatment group had any additional requirement of therapy.

**Table 2:** Number of patients receiving different doses of drugs at successive visits

Visit	Drug Dose (mg)	Pregabalin Group (N=31)	Sertraline Group (N=31)	Total Patients
1st	25	31	31	62
2nd	25	1	1	2
	50	30	30	60
3rd	50	31	31	62

As shown in Table 3, before drug initiation (Visit 1), the mean 5D itch scale scores in the pregabalin and sertraline groups were 15.74±3.62 and 15.22±4.35, respectively, which implies that no significant difference was seen for the 5D itch scale score between two study groups (p-value=0.614). After two weeks of drug administration (Visit 2), the mean 5D itch scale scores in pregabalin and sertraline groups were 11.58±2.90 and 12.51±4.03, respectively. At this point, no significant difference was seen for the 5D itch scale score between study drugs (p-value=0.299). Similarly, after four weeks of drug administration (Visit 3), the mean 5D itch scale scores in the pregabalin and sertraline groups were 9.09±2.89 and 10.54±3.17, respectively. At this point again, no significant difference was seen for the 5D itch scale score between study drugs (p-value=0.064).

**Table 3:** 5D itch scale score in study groups A p-value of less than 0.05 is considered statistically significant.

Variables	Statistics	Pregabalin Group	Sertraline Group	P-value
Visit 1	N	31	31	0.614
	Mean	15.74	15.22	
	SD	3.62	4.35	
	Minimum	9	9	
	Maximum	23	24	
Visit 2	N	31	31	0.299
	Mean	11.58	12.51	
	SD	2.9	4.03	
	Minimum	7	7	
	Maximum	19	23	
Visit 3	N	31	31	0.064
	Mean	9.09	10.54	
	SD	2.89	3.17	
	Minimum	6	6	
	Maximum	21	16	

As shown in Table 4, before drug initiation (first visit), four (12.9%) patients in the pregabalin group and seven (22.6%) patients in the sertraline group had severe pruritus, while 11 (35.5%) patients in pregabalin and 15 (48.4%) patients in sertraline had mild pruritus (p-value=0.313). On the second visit, 15 (48.4%) patients in the pregabalin group and 14 (45.2%) patients in the sertraline group had mild pruritus, while 11 (35.5%) patients in pregabalin and seven (22.6%) patients in the sertraline group had no pruritus. No significant difference was seen for the intensity of pruritus between groups at Visit 2 (p-value=0.306). On the third visit, 18 (58.1%) patients in the pregabalin group and 16 (51.6%) patients in the sertraline group had no pruritus. Thus, no significant difference was seen for the intensity of pruritus between groups at Visit 3 (p-value=0.249).

Meanwhile, the pregabalin group revealed significant improvement (p-value<0.00001) in itching intensity during follow-up (Visit 2 and Visit 3) as compared to the pre-medication pregabalin group (Visit 1), as shown in Table 4. Furthermore, the itching intensity was significantly reduced at each follow-up as measured by Visit 1 versus Visit 2 (p<0.0001) and Visit 2 versus Visit 3 (p=0.001). Similarly, as shown in Table 4, the sertraline group revealed significant improvement (p-value=0.00003) in itching intensity during follow-up (Visit 2 and Visit 3) as compared to the pre-medication sertraline group (Visit 1). Furthermore, the itching intensity has significantly reduced at each follow-up as measured by Visit 1 verses Visit 2 (p=0.01) and Visit 2 verses Visit 3 (p=0.03).

**Table 4:** Intensity of pruritus in the study groups on successive visits A p-value of less than 0.05 is considered statistically significant.

Variables	Intensity of Pruritus	Pregabalin Group (N= 31)	Sertraline Group (N=31)	P-Value
Visit 1	Severe	4	7	0.313
	Moderate	15	8	
	Mild	11	15	
	No Pruritus	1	1	
Visit 2	Severe	0	2	0.306
	Moderate	5	8	
	Mild	15	14	
	No Pruritus	11	7	
Visit 3	Severe	0	0	0.249
	Moderate	1	3	
	Mild	12	12	
	No Pruritus	18	16	

## Discussion

In recent years, there has been an upsurge in the modes of treatment for UP patients. Moisturizers, topical calcineurin inhibitors, phototherapy, antihistamines, high-quality dialysis, serotonin and opioid receptor agonists/antagonists, mast cell stabilizers, montelukast, activated charcoal, etc. have been employed to improve the intensity of itching. In 2021, the United States Food and Drug Administration (FDA) approved difelikefalin (intravenous) for the first time as the medication for UP [19]. Thus far, there have been several oral medicines under prescription for UP patients. Among these, our research compares the efficacy of two medications, pregabalin and sertraline, which are key modulators of neurotransmitters: possibly acting by decreasing neurotransmitter release. We researched and compared these drugs as these are easily available and cost-effective, and the oral route is the most convenient form for drug administration particularly in under-resource countries such as Pakistan.

The research has found that there were no significant differences in 5D itching score outcomes between the two experiment groups: pregabalin and sertraline. The itching scores among the two groups were similar before drug initiation at the first visit (15.74 vs. 15.22, p-value=0.614). After treatments, the 5D score was 11.58 vs. 12.51 (p-value=0.299) on the second visit and 9.09 vs. 10.54 (p-value=0.064) on the third visit. Similar findings were observed for the intensity domains of pruritus in two study groups. No pruritus domains at the first visit were 3.2% vs. 3.2% (p-value=0.313), 35.5% vs. 22.6% (p-value=0.306) at the second visit, and 58.1% vs. 51.6% (p-value=0.249) at the third visit. Moreover, the level of pruritus was comparable across the two groups. The 5D itch scale score was lower in the pregabalin group, although the difference was not statistically significant. Overall, no significant difference was observed in this research while comparing two treatment groups at any time throughout the research period on UP intensity.

Our research findings contrast with the well-documented fact that pregabalin is a better choice as compared to other drugs to treat UP patients. For instance, in a head-to-head comparison of pregabalin with sertraline for the treatment of pruritus, it was found that pregabalin was more effective and had fewer negative side effects [8]. In our research, side effects were however not measured, which is a limitation of this study. Other clinical studies have mentioned that the antiepileptic medication - pregabalin - may be helpful if patients are suffering from convulsions [20,21]. Additionally, pregabalin has been shown more effective in reducing the severity of pruritus for the treatment of pruritus associated with dialysis, a recent trial comparing pregabalin and gabapentin came to the same conclusion [22]. It has been demonstrated that pruritus was relieved in 85% of patients out of 71 by both of these medications and further suggested that patients sensitive to gabapentin might tolerate pregabalin [23]. Similarly, pregabalin daily improved pruritus and neuropathic pain in both of the two drug groups [24]. The adverse reaction of gabapentin (e.g., drowsiness, dizziness, and somnolence) was higher as compared to pregabalin but not statistically significant [25]. However, a systemic review concluded that gabapentin is overall the best option as a treatment for UP [26].

Despite this, little research has emphasized sertraline, a selective serotonin reuptake inhibitor (SSRI), which significantly lessened the intensity of pruritus [15,27-29]. For instance, 20 CKD-associated pruritus patients who were not on dialysis were given sertraline after failing antihistamine medication among which these patients reported improvement in itching 5.1 weeks after treatment [15]. Similarly, sertraline-treated individuals in a double-blind, randomized controlled trial with 50 patients who reported much less pruritus than placebo 57 treated patients in four weeks of therapy [27]. In addition, it was discovered that sertraline was effective in lowering the severity of UP in CKD patients. Ten (52.6%) of the 19 individuals who participated in a trial experienced severe pruritus. At the end of the research, just two (10.7%) of them remained affected with severe pruritus after the treatment [28]. Even though sertraline is less often used because of the time it takes for the effect, it may be useful for patients who are suffering from depression. Patients with CKD and pruritus have shown considerable improvement in itching when given sertraline, according to two trials [15,29]. There are further advantages that sertraline may confer over competing medications. Patients with moderate to severe pruritus have been shown to have an increased risk of developing depression, and sertraline's primary function is antidepressant [27].

## Conclusions

It is concluded that no significant difference was observed in the efficacy between pregabalin and sertraline therapy for UP patients under maintenance hemodialysis. Meanwhile, both pregabalin and sertraline significantly reduced itching intensity in each treatment group. In each domain of the 5D pruritus scale, a significant difference was found in post follow-up of pregabalin and sertraline therapy.

## References

[1] Silva P, Mohebbi N (2022). Kidney metabolism and acid-base control: back to the basics. European Journal of Physiology.

[2] Webster A, Nagler E, Morton R (2017). Chronic kidney disease. Lancet.

[3] Kazmi W (2013). A greater than expected prevalence of chronic kidney disease (CKD) in Pakistan. Annals Abbasi Shaheed Hospital and Karachi Medical & Dental College.

[4] Kazancioğlu R (2013). Risk factors for chronic kidney disease: an update. Kidney Int Suppl (2011).

[5] Verduzco HA, Shirazian S (2020). CKD-associated pruritus: new insights into diagnosis, pathogenesis, and management. Kidney Int Rep.

[6] Lowe M, Jervis S, Payton A, Poulton K, Worthington J, Gemmell I, Verma A (2022). Systematic review of associations between HLA and renal function. Int J Immunogenet.

[7] Pakfetrat M, Basiri F, Malekmakan L, Roozbeh J (2014). Effects of turmeric on uremic pruritus in end stage renal disease patients: a double-blind randomized clinical trial. J Nephrol.

[8] Wahab A, Mohammad A, Anber N (2016). Sertraline versus pregabalin in treatment of pruritus in maintenance hemodialysis patients: a single
center prospective, cross-over study. AJOD.

[9] Khan TM, Alhafez AA, Syed Sulaiman SA, Bin Chia DW (2015). Safety of pregabalin among hemodialysis patients suffering from uremic pruritus. Saudi Pharm J.

[10] Foroutan N, Etminan A, Nikvarz N, Shojai Shahrokh Abadi M (2017). Comparison of pregabalin with doxepin in the management of uremic pruritus: a randomized single blind clinical trial. Hemodial Int.

[11] Yue J, Jiao S, Xiao Y, Ren W, Zhao T, Meng J (2015). Comparison of pregabalin with ondansetron in treatment of uraemic pruritus in dialysis patients: a prospective, randomized, double-blind study. Int Urol Nephrol.

[12] Gajraj NM (2007). Pregabalin: its pharmacology and use in pain management. Anesth Analg.

[13] MacQueen G, Born L, Steiner M (2001). The selective serotonin reuptake inhibitor sertraline: its profile and use in psychiatric disorders. CNS Drug Rev.

[14] Mahmoud O, Oladipo O, Mahmoud RH, Yosipovitch G (2023). Itch: from the skin to the brain - peripheral and central neural sensitization in chronic itch. Front Mol Neurosci.

[15] Chan KY, Li CW, Wong H, Yip T, Chan ML, Cheng HW, Sham MK (2013). Use of sertraline for antihistamine-refractory uremic pruritus in renal palliative care patients. J Palliat Med.

[16] Kubanek A, Paul P, Przybylak M (2021). Use of sertraline in hemodialysis patients. Medicina (Kaunas).

[17] Nagler EV, Webster AC, Vanholder R, Zoccali C (2012). Antidepressants for depression in stage 3-5 chronic kidney disease: a systematic review of pharmacokinetics, efficacy and safety with recommendations by European Renal Best Practice (ERBP). Nephrol Dial Transplant.

[18] Elman S, Hynan LS, Gabriel V, Mayo MJ (2010). The 5-D itch scale: a new measure of pruritus. Br J Dermatol.

[19] (2024). Novel drug approvals for 2021. https://www.fda.gov/drugs/novel-drug-approvals-fda/novel-drug-approvals-2021.

[20] Ahuja RB, Gupta GK (2013). A four arm, double blind, randomized and placebo controlled study of pregabalin in the management of post-burn pruritus. Burns.

[21] Shavit L, Grenader T, Lifschitz M, Slotki I (2013). Use of pregabalin in the management of chronic uremic pruritus. J Pain Symptom Manage.

[22] Ravindran A, Kunnath RP, Sunny A, Vimal B (2020). Comparison of safety and efficacy of pregabalin versus gabapentin for the treatment of uremic pruritus in patients with chronic kidney disease on maintenance haemodialysis. Indian J Palliat Care.

[23] Rayner H, Baharani J, Smith S, Suresh V, Dasgupta I (2012). Uraemic pruritus: relief of itching by gabapentin and pregabalin. Nephron Clin Pract.

[24] Solak Y, Biyik Z, Atalay H (2012). Pregabalin versus gabapentin in the treatment of neuropathic pruritus in maintenance haemodialysis patients: a prospective, crossover study. Nephrology (Carlton).

[25] Eusebio-Alpapara KM, Castillo RL, Dofitas BL (2020). Gabapentin for uremic pruritus: a systematic review of randomized controlled trials. Int J Dermatol.

[26] Simonsen E, Komenda P, Lerner B (2017). Treatment of uremic pruritus: a systematic review. Am J Kidney Dis.

[27] Pakfetrat M, Malekmakan L, Hashemi N, Tadayon T (2018). Sertraline can reduce uremic pruritus in hemodialysis patient: a double blind randomized clinical trial from Southern Iran. Hemodial Int.

[28] Shakiba M, Sanadgol H, Azmoude HR (2012). Effect of sertraline on uremic pruritus improvement in ESRD patients. Int J Nephrol.

[29] Mayo MJ, Handem I, Saldana S, Jacobe H, Getachew Y, Rush AJ (2007). Sertraline as a first-line treatment for cholestatic pruritus. Hepatology.

